# Prognostic Markers in Chondrosarcoma: Evaluation of Cell Proliferation and of Regulators of the Cell Cycle

**DOI:** 10.1080/13577149778344

**Published:** 1997-06

**Authors:** Sean P. Scully, Lester J. Layfield, John M. Harrelson

**Affiliations:** Section of Musculoskeletal Oncology Duke University Medical Center PO Box 3312 Durham NC 27710 USA

## Abstract

*Purpose*. The prognosis, treatment principles and prediction of clinical outcome of patients with chondrosarcoma currently rest on histologic grading which is somewhat ambiguous due to difficulty in pathologic interpretation of this neoplasm. Immunohistochemistry, flow cytometry and oncogene/tumor suppressor gene expression have been examined as alternative indices to predict the biologic behavior of these tumors. Because of partial successes obtained with flow cytometry and because of the improvement in predicting recurrence offered by examining the S-phase fraction, we undertook the current study to determine if expression of specific regulators of the cell cycle would act as prognostic indicators for these patients.

*Subjects/methods*. We examined archival pathologic specimens from 39 patients with at least 2 years' clinical follow-up for the presence of p53, Rb, *src* and MIB-1 by immunohistochemistry and correlated this with clinical histories and incidence of recurrence.

*Results*. While Rb, p53 and *src* gene products were identified to a variable extent in these specimens, there was no prognostic significance to their expression. In contrast, MIB-1, an epitope expressed only during semiconservative replication and an accepted marker of cell proliferation, served as a significant prognostic indicator. MIB-1 staining was present in 14.5% of tumor cells in all specimens (range 0–59%). When MIB-1 staining was examined with respect to disease recurrence, there was a statistically significant association between staining and histologic grade (*p* < 0.05) as well as event-free survival (*p* < 0.02). Comparing survival curves stratified by MIB-1 expression, there was a significant decrease in event-free survival associated with increasing MIB-1 indices (*p* < 0.003). Covariates that were associated with event-free survival include histologic grade (*p* = 0.025) and stage (Musculoskeletal Tumor Society) (*p* = 0.014). There was no statistical association with patient age (*p* = 0.15), tumor size (*p* = 0.47), tumor histology (*p* = 0.62) or anatomic location (*p* = 0.316).

*Discussion*. These results indicate that determination of the proliferation index by MIB-1 immunostaining may serve as a useful adjunct to current histopathologic classification. Patients with a high proliferation index may benefit from established adjuvant therapies or experimental approaches including immunotherapy or biologic modulation.


*Sarcoma* (1997) 1, 79–87

ORIGINAL ARTICLE

### Prognostic markers in chondrosarcoma: evaluation of cell proliferation and of regulators of the cell cycle

SEAN P. SCULLY, LESTER J. LAYFIELD & JOHN M. HARRELSON

*Section of Musculoskeletal Oncology, Duke University Medical Center, Durham, USA*

**Abstract**

*Purpose.* The prognosis, treatment principles and prediction of clinical outcome of patients with chondrosarcoma currently rest on histologic grading which is somewhat ambiguous due to difficulty in pathologic interpretation of this neoplasm. Immunohistochemistry, flow cytometry and oncogene/tumor suppressor gene expression have been examined as alternative indices to predict the biologic behavior of these tumors. Because of partial successes obtained with flow cytometry and because of the improvement in predicting recurrence offered by examining the S-phase fraction, we undertook the current study to determine if expression of specific regulators of the cell cycle would act as prognostic indicators for these patients.

*Subjects/methods.* We examined archival pathologic specimens from 39 patients with at least 2 years' clinical follow-up for the presence of p53, Rb, *src* and MIB-1 by immunohistochemistry and correlated this with clinical histories and incidence of recurrence.

*Results.* While Rb, p53 and *src* gene products were identified to a variable extent in these specimens, there was no prognostic significance to their expression. In contrast, MIB-1, an epitope expressed only during semiconservative replication and an accepted marker of cell proliferation, served as a significant prognostic indicator. MIB-1 staining was present in 14.5% of tumor cells in all specimens (range 0–59%). When MIB-1 staining was examined with respect to disease recurrence, there was a statistically significant association between staining and histologic grade ($p < 0.05$) as well as event-free survival ($p < 0.02$). Comparing survival curves stratified by MIB-1 expression, there was a significant decrease in event-free survival associated with increasing MIB-1 indices ($p < 0.003$). Covariates that were associated with event-free survival include histologic grade ($p = 0.025$) and stage (Musculoskeletal Tumor Society) ($p = 0.014$). There was no statistical association with patient age ($p = 0.15$), tumor size ($p = 0.47$), tumor histology ($p = 0.62$) or anatomic location ($p = 0.316$).

*Discussion.* These results indicate that determination of the proliferation index by MIB-1 immunostaining may serve as a useful adjunct to current histopathologic classification. Patients with a high proliferation index may benefit from established adjuvant therapies or experimental approaches including immunotherapy or biologic modulation.

**Key words:** *chondrosarcoma, prognostic indicators, cell proliferation index.*

### Introduction

Chondrosarcoma remains a challenge for all disciplines of oncology. Histologic grading systems have traditionally been used for prediction of clinical behavior.[1] However, the pathologist is not infrequently humbled in an attempt to distinguish a benign cartilaginous lesion from a malignant one. The radiation therapist and the medical oncologist have been unable to provide any significant advances in local or systemic adjuvant treatment.[2] The patient can be confronted with either local or systemic recurrence for as long as 10 years after the primary resection. Taken in conjunction, these factors highlight the need to predict which patients are at highest risk to fail, and the need for novel approaches to systemic adjuvant therapies.

Surgical resection remains the primary modality for the treatment of chondrosarcoma and survival is most directly related to the adequacy of the surgical procedure.[3,4] Patients with low-grade chondrosarcoma that are adequately treated exhibit 5-year survival rates that approach 90%.[5] In contrast, those with inadequate surgical treatment and those with high-grade lesions faired much more poorly with 5-year survival rates of 43%.[6–8]

Several lines of evidence suggest that increased rates of cell proliferation are associated with poor prognosis in patients with chondrosarcoma. These include alterations in mitotic index, flow cytometry determined by ploidy and S-phase, Ki-67 staining and oncogene expression which have been identified in these patients. We undertook the current study

Correspondence to: S. P. Scully, Section of Musculoskeletal Oncology, Duke University Medical Center, PO Box 3312, Durham, NC 27710, USA. Fax: +1 919 681 7645; E-mail: scull002@MC.Duke.edu.

1357-714X/97/020079-09 $9.00 ©1997 Carfax Publishing Ltd in an effort to compare clinical outcome and histologic grade with determination of *src*, Rb, p53 and MIB-1 markers determined by immunohistochemical analysis. These markers were selected because they either reflect or in part control the proliferative activity within the cell cycle. MIB-1 (Ki-67) is expressed in all phases of the cell cycle except for $G_0$ and some portions of $G_1$. As such, it represents a good marker of the overall proliferative activity in a population of neoplastic cells. p53 is thought to act as a rate limiting protein affects cell passage through the $G_1$ phase of the cell cycle. Hence, its absence or a mutation may lead to a transformed and non-functional phenotype. While the native form of p53 is short lived, the mutant forms are thought to persist and be detectable by immunostaining. The p53 gene is located on chromosome 17 and appears to cause cell arrest in the $G_1$ phase.[9–11] This protein, the deletion of both wild-type alleles or the presence of a dominant mutation in one allele can lead to unregulated cell replication. Several mutations of the p53 gene are known and are associated with defective protein products. These protein products lack the normal proliferation-suppressing properties and have prolonged half-lives. The prolonged half-life allows accumulation of the protein and its detection in abnormally large amounts by quantitative immunohistochemistry.[12]

The *src* gene product is a 60-kDa phosphoprotein located on the cytoplasmic side of the plasma membrane that functions as a tyrosine kinase. It has been implicated in the development of avian sarcomas and may act through phosphorylation of cytoskeletal proteins such as vinculin. The *src* oncogene produces a protein product which, along with *ras* and the *myc* oncogenes, affect cell proliferation and DNA synthesis.[13] The serine/threonine protein kinase p34 is the major regulator of progression through the mammalian cell cycle and it appears to activate *src*.[14] The precise role of *src* is unknown but it may be associated with cytoskeletal alterations which occur at mitosis and is required for cell division.[15]

Rb-1 is a nuclear protein which is thought to act as a tumor suppressor gene. This is based on an absence of expression in retinoblastoma and the ability to restore a normal phenotype to osteosarcoma cells with transfection of this gene. The role of this gene in the etiology of chondrosarcoma is unknown. The RB (RB-1) gene encodes for a protein, $p105^{RB}$, which has homology with a region of protein p107. p107 is known to bind to the T antigen on E1A.[16] $pl05^{RB}$ is known to bind with double-stranded DNA in a non-sequence-specific manner and appears to inhibit the stimulation of DNA polymerase $\delta$. $p105^{RB}$ also appears to repress *myc* transcription in some tumor cells.[14] Hence, RB appears to be an important suppressor gene for the cell cycle.

This report correlates markers of cell cycle abnormalities in patients diagnosed with chondrosarcoma with histologic grading and vigilant clinical follow-up. The 39 patients had adequate archival pathology specimen, non-metastatic disease at the time of presentation and were treated at a single institution by a single surgeon over a 21-year period. All patients underwent resection with negative margins indicating adequate initial therapy. The data suggest that similar to histologic grading, proliferation index derived from MIB-1 staining was a significant prognostic indicator of clinical outcome in these patients.

### Subjects and methods

### *Subjects*

Sixty-six patients were treated at Duke University Medical Center between 1972 and 1993 for primary non-metastatic chondrosarcoma. Of these, 39 had adequate clinical history and sufficient pathologic material available to permit evaluation of immunohistochemical markers for prognostic significance. Within this group of 39 patients, biopsies were performed and clinical staging completed with radiographs, radionucleotide, computed tomography and magnetic resonance imaging scans when available. The patients each underwent a wide or radical resection depending on the specifics of the clinical situation and this was carried out by a single surgeon. All surgical margins were negative indicating adequate initial treatment. The patients were then followed with clinical examination and serial radiographs for an average of 6.7 years with a minimum follow-up of 24 months. There were two patients who presented with local failures and seven patients who presented with distant failures. Both patients with local recurrences were patients who subsequently developed distant disease. Three patients received radiation therapy and seven received chemotherapy post-operatively following either local or distant recurrence. All patients with distant failure succumbed to the disease process.

### *Archival specimens*

Surgical specimens obtained at the time of initial resection were routinely fixed in 10% neutral buffered formalin and subsequently paraffin embedded. The histologic sections for the patients included in this study were obtained from the appropriate paraffin blocks and were reviewed by one pathologist (LJL).

### *Immunohistochemistry*

Immunohistochemistry was performed in the Duke University Medical Center Comprehensive Cancer Facility as previously described.[14,17–19] Histologic material from all cases was examined by a single pathologist for sample adequacy. Upon tissue block retrieval, serial 5 μm sections were mounted on Fisher + Plus + slides (Fischer, Charlotte, NC, USA). The slides were dried overnight at 65°Cin an Imperial oven (Baxter, McGraw Park, IL, USA). Specimens were deparaffinized in three successive xylene baths and were cleared in absolute ethanol. Slides were then gradually brought to hydration. All antibodies were diluted in phosphate-buffered saline with 2% bovine serum albumin (Sigma Chemical Co., St Louis, MO, USA), pH 7.4 (PBS/BSA). As negative controls, murine $IgG_1$ (Coulter Source, Marietta, GA, USA) was diluted 1:100 and normal rabbit serum (Grand Island Biochemical Co., Grand Island, NY, USA) was diluted 1:1000 in 2% PBS/BSA.

*MIB-1.* The primary antibody used tor MIB-1 determination is a murine monoclonal $IgG_1$ that reacts with the Ki-67 nuclear antigen (345- and 395-kDa double band in Western blot analysis) expressed by proliferating cells.[20] Deparaffinized slides were incubated in 10 mM citrate buffer, pH 6.0 (Sigma) for antigen retrieval. Antigen retrieval was carried out using a 700-W microwave (Quasar, model MQ7677BW, Elk Grove, IL, USA) as recommended by the antibody manufacturer (Immunotech). Slides were placed in 10 mM citrate solution, pH 6.0, and microwaved at full power for 5 min. They were then allowed to cool and microwaved again for 5 min and were slowly brought to water, trying to avoid any d*ras*tic temperature change. Assay slides were rinsed with PBS and then placed in 5% normal goat serum (NGtS) for 20 min. Excess NGtS was blotted from the slides and the primary antibodies ($IgG_1$ , MIB-1) diluted 1:100 were applied and incubated overnight at 4°C for approximately 18 h. All of the assay incubations were carried out in a humidity chamber to prevent solution evaporation. After bringing the slides to room temperature, they were rinsed in PBS, three times for 5 min, and incubated with goat anti-mouse biotinylated antibody (BioGenex, San Ramon, CA, USA) for 35 min. The slides were rinsed in PBS three times for 5 min followed by application of peroxidase-conjugated strepavidin label. The slides were then developed for 5 min with chromagen 3,3′diaminobenzidene (Sigma) (0.5% diaminobenzidine in 0.05 M Tris buffer and 0.6% hydrogen peroxide). Finally, the assay slides were rinsed in running tap water for 10 min, counter-stained with 1% methyl green (Sigma), dehydrated in acetone and cover-slipped in Protexx (Baxter).

*Rb, p53, src.* Primary antibody directed against p53 (PAb 1801) was obtained from Oncogene Science, Manhasset, NY, USA (Ab-2) which is an affinity-purified $IgG_1$ monoclonal antibody that recognizes a denaturation-resistant epitope in the human p53 protein located between amino acids 32 and 79.[21] Immunostaining was performed with 1.0 $\mu g\ ml^{-1}$ of primary antibody on deparaffinized slides. The secondary antibody was biotinylated affinity-purified horse anti-mouse IgG (Vector). Sections were developed in diaminobenzidine solution, rinsed and counter-stained in 1% methyl green in sodium acetate buffer, pH 5.2.

The antibody to *src* is a purified IgG (Oncogene Science, Uniondale, NY, USA). The antibody (titer of 1:100) was prepared and run in a fashion described for MIB-1. The antibody directed against Rb protein product was obtained from Oncogene Science (Uniondale, NY, USA) and run as just described using a titer of 1:50.

The quantitation of immunostaining for each of the markers was determined using a CAS 200 Image Analysis System (Becton Dickinson Cellular Imaging Systems, San Jose, CA, USA) in combination with the Quantitative Proliferation Index CAS Software Program (Becton Dickinson Cellular Imaging Systems). Quantitation of proliferative index has been described previously for tumor tissue sections as the percentage of total nuclear area that stains positively with antibody. A similar approach was applied for p53 and Rb.[22] Data obtained by computerized static image analysis for src were expressed as a percentage of cellular area positively stained with monoclonal antibody relative to the total cellular area. Fifteen fields of the tumor were analyzed at a magnification of ×400. The automated mean for staining was scored as the representative index of the neoplasm or it's components. Control sections, stained with normal murine $IgG_1$ were prepared in each case, observed by standard microscopy and analyzed to establish background immunostaining thresholds. Intra-observer reproducibility was determined as described previously.[23]

### *Statistical analysis*

Patients were divided into low and high expression groups based on the median expression value for each epitope. For MIB-1 this was $> 6.25\%$, for Rb, *src* and p53 $> 2\%$. The segregation into low and high expression was then used to examine the correlation of these parameters with clinical outcome.

All statistical tests were two-sided with $a = 0.05$. The analysis of variance test with Tukey's honest significant difference procedure or two sample *t*-test were performed to assess the association of MIB-1 with other prognostic factors. The Kaplan–Meier[24] procedure was used to determine survival curves and the curves were subsequently compared using the Wilcoxon rank sum test.[25] $\chi^2$ tests were used to test for association of survival time with covariates. Parametric tests were supplemented by non-parametric analysis (Wilcoxon rank sum test, Kruskal–Wallis test) which yielded similar results.

**Table 1.** *Immunostaining and prognostic significance*

| Parameter | MIB-1 | Rb | *src* | p53 |
|---|---|---|---|---|
| Qualitative staining | 35/39 | 24/39 | 19/39 | 16/39 |
| Quantitative staining | 14.5% | 4.3% | 2.6% | 4.7% |
| Prognostic significance | $p < 0.01$ | $p = 0.49$ | $p = 0.62$ | $p = 0.67$ |

The immunostaining is presented as the percentage of patients who demonstrated positive staining for the epitope and also quantitatively as the percentage of cellular area with positive staining normalized to total cellular area. The prognostic significance was determined by survival analysis.

### Results

Twenty-seven patients underwent a primary attempt at limb salvage while 12 patients underwent a primary amputation, all with wide or radical surgical margins. There were two patients who developed a local recurrence and were treated with radiation therapy. Chemotherapy was administered in the seven patients who developed distant metastasis. Both of the patients who developed local recurrences also developed distant metastasis and expired.

### *Immunostaining*

The qualitative results of immunostaining of chondrosarcoma specimens for the cell cycle regulators, p53, *src* and Rb, and for the proliferation index and their prognostic significance are summarized in Table 1. The specimens were further studied by quantitative image analysis in an attempt to make the evaluation similar to that of the cell proliferation index. There was no statistically significant correlation between immunostaining results for Rb, p53 and *src* with histologic type, grade or stage in these patients.

Twenty-four of the 39 patients demonstrated positive staining with the Rb antibody (range 0–23% with a median of 4.3%). Survival analysis indicated that there was no prognostic significance to Rb expression, $p = 0.49$ (Fig. 1). Nineteen of 39 patients demonstrated a positive immunostaining with the *src* antibody (range 0–20% with a median of 2.6%). Kaplan–Meier survival analysis indicated that the survival in low *src* expression patients was equivalent to that in patients expressing higher amounts of the gene product (Fig. 2). Wilcoxon rank sum analysis indicates that this difference in event-free survival is not statistically significant, $p = 0.62$. Sixteen of 39 patients demonstrated an abnormality in p53 structure on the basis of immunostaining (range 0–47% with a median of 4.7%). Patients with p53 abnormalities demonstrated a shorter event-free survival than those with undetectable p53; however, this difference was not statistically significant, $p = 0.67$ (Fig. 3). However, it should be noted that in four of the six patients with a dedifferentiated chondrosarcoma, strong p53 staining was observed.

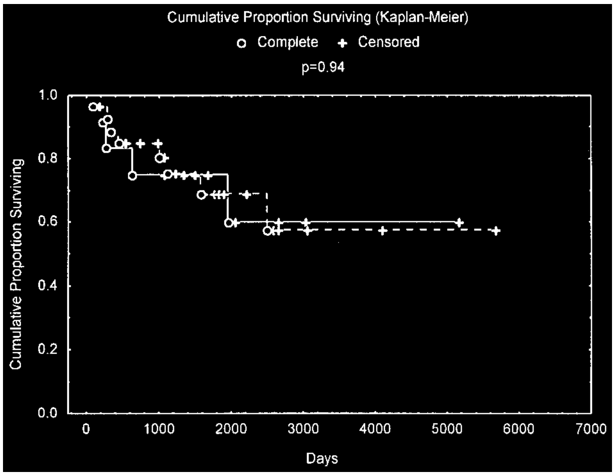

**Fig. 1.** *Survival of patients with chondrosarcoma analyzed by quantitative immunohistochemical staining for the Rb antigen. Low Rb staining (solid line) constituted those patients with less than 2% of cellular area staining positively with respect to total cellular area, while those with >2% constituted the high group (dotted line).*

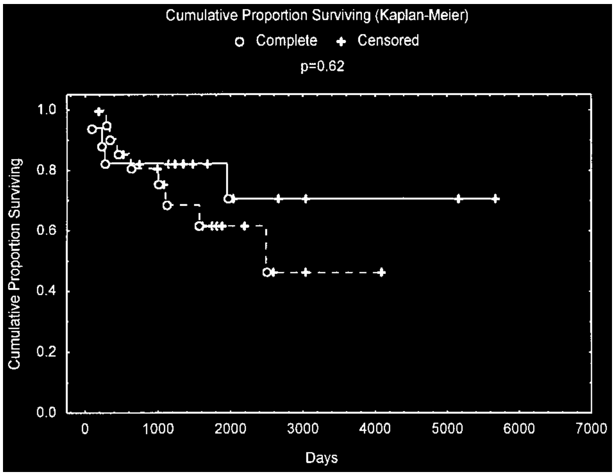

**Fig. 2.** *Survival of patients with chondrosarcoma analyzed by quantitative immunohistochemical staining for the* src *antigen. Low* src *staining (solid line) constituted those patients with less than 2% of cellular area staining positively with respect to total cellular area, while those with >2% constituted the high group (dotted line).*

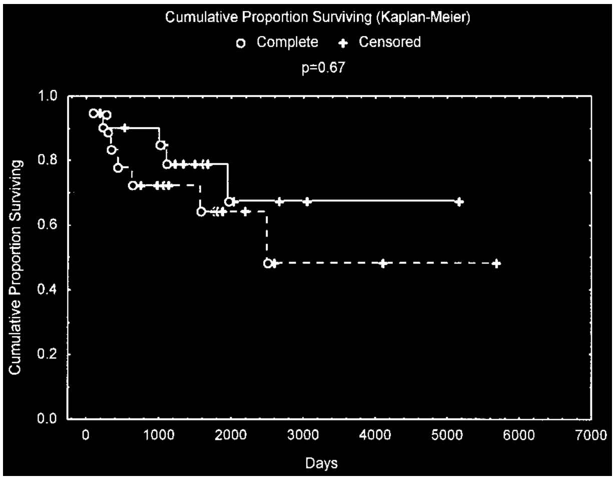

**Fig. 3.** *Survival of patients with chondrosarcoma analyzed by quantitative immunohistochemical staining for the p53 antigen. Low p53 staining (solid line) constituted those patients with less than 2% of cellular area staining positively with respect to total cellular area, while those with >2% constituted the high group (dotted line).*

MIB-1 immunostaining was used to quantitate a proliferation index. The mean of the proliferative index was 14.5% with a range of 0–59%. There was a statistically significant association between MIB-1 level and histologic grade, $p < 0.05$ (Table 2), and with disease status, $p < 0.02$ (Table 3). Comparing survival curves stratified by MIB-1 staining, there was a significant decrease in survival with increasing MIB-1 indices ($p = 0.003$; Fig. 4). Cox analysis was performed and the proliferation index was an independent predictor of event-free survival. Covariates that were associated with survival time included histologic grade ($p = 0.025$) and Enneking stage ($p = 0.0142$). There was no association with age ($p = 0.1556$), tumor size ($p = 0.4671$), tumor type ($p = 0.6211$) or anatomic location ($p = 0.3160$).

The mean proliferation index was examined with respect to histologic type of chondrosarcoma. There were 22 classic intramedullary chondrosarcomas, six dedifferentiated chondrosarcomas and 11 chondrosarcomas including those arising in osteochondromas (extraskeletal, clear cell and myxoid variants were grouped to facilitate comparison). The mean proliferation index for the intramedullary chondrosarcomas was 0.106, that for the dedifferentiated chondrosarcomas was 0.396 and that for the grouped variants was 0.086 (Table 4). The dedifferentiated chondrosarcomas had a proliferation index higher than the mean index and also had a lower event-free survival; however, survival analysis could not be performed due to limited numbers.

### Discussion

Neoplastic cell proliferation, invasion and metastases represent independent and important variables determining the aggressiveness and behavior of neoplastic cell populations. Assessment of molecular or morphologic markers for these properties of neoplastic cell populations would be helpful for predicting the biological behavior of human malignancies. Traditionally, such markers have been assessed by morphologic analysis of mitotic figure counts,

**Table 2.** *Proliferation index and histologic grade*

| Histologic grade | Proliferation index Mean | Proliferation index SE | No. of patients |
|---|---|---|---|
| 1 | 8.14 | 2.8 | 14 |
| 2 | 7.00 | 1.7 | 16 |
| 3 | 37.8 | 7 | 9 |

The correlations between histologic grade and cell proliferation index are displayed. Histologic grade was determined following resection of the tumor. The mean of the proliferation index and the standard error (SE) of the mean are indicated. The number of patients for each group is also indicated.

**Table 3.** *Proliferation index and event-free survival*

| Survival | Proliferation index Mean | Proliferation index SE | No. of patients |
|---|---|---|---|
| Disease free | 11.3 | 17.2 | 32 |
| Recurrence | 29.2 | 15.8 | 7 |

$p < 0.0242$.

The proliferation index is examined for patients who remained disease free and for those who experienced a recurrence. The mean and the standard error (SE) of the mean for proliferation indices are displayed. The number of patients in each group is also indicated.

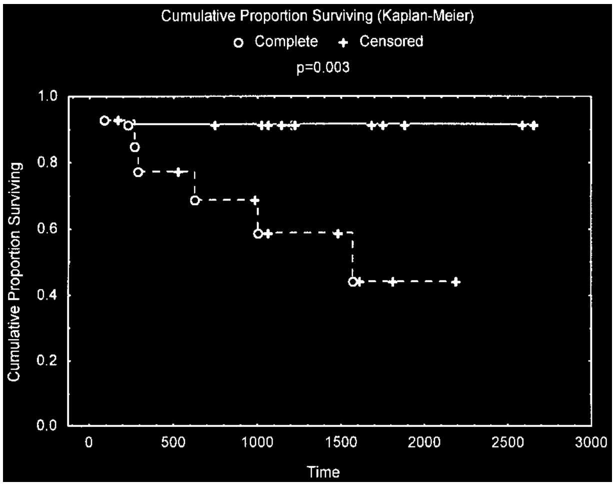

**Fig. 4.** *Survival of patients with chondrosarcoma analyzed by quantitative immunohistochemical staining for the MIB-1 antigen. Low MIB-1 staining (solid line) constituted those patients with less than 6.25% of cellular area staining positively with respect to total cellular area, while those with >6.25% constituted the high group (dotted line)*

**Table 4.** *Proliferation index and histologic tumor type*

| Histologic type | Proliferation index | | No. of patients |
|---|---|---|---|
| | Mean | SE | |
| Intramedullary | 10.6 | 2.9 | 22 |
| Dedifferentiated | 39.6 | 7.8 | 6 |
| Other | 8.6 | 4.3 | 11 |

The proliferation index for each of the histologic subtypes of chondrosarcoma is displayed. The proliferation index is displayed as the mean and the standard error (SE) of the mean for each group. The number of patients with each diagnosis is also displayed.

vascular invasion and histologic analysis of host/tumor boundary interactions. Recently, a variety of oncogenes and their protein products have been recognized as regulators of cell behavior. A variety of these protein products are known to affect the cell cycle and hence represent control substances for cell proliferation. Others like MIB-l appear to be useful as markers of overall cell proliferation. The regulation of cell proliferation by oncogene protein products is complex and at present is only partially understood. We chose to study the predictive value of the cell proliferation index as determined by MIB-1 as well as the predictive value of quantitation of certain oncogene products known to have regulatory functions in the cell proliferation cycle.

### *src*

It is known that cells transformed by *src* will be inhibited from entering into the S-phase when anti-*ras* antibodies are injected into cell cultures. Such observations suggest that *src* oncogene transforms cells via a pathway involving *ras*. Other tumor suppressor genes including p53 and RB-1 activate pathways that converge downstream of the *ras* oncogene point of control. The *src* family of genes codes for proteins with tyrosine kinase activity.[26] This activity is normally inhibited *in vivo* by phosphorylation of Tyr 527. In intact cell, the targets of *src* involved in regulating cell proliferation have not been identified[27] but *src* may play an indirect role in cell cycle regulation by its ability to phosphorylate p34.[28] In studies with mice, *src* appears to be essential for bone formation, thus implicating a physiologic role in normal mesenchymal tissue. Early reports had implicated *myc* and *src* oncogenes in the proliferation and synthesis of specific matrix components in chondroblasts.[29] Zhu *et al.* were unable to detect *src* gene transcription using Northern hybridization.[30] We were able to identify 19 of 39 patients with immunohistochemical evidence of *src* gene product translation but there was no statistical correlation with clinical outcome. We conclude that the role of the *src* gene product in the etiology and clinical behavior of chondrosarcoma is currently unknown but is probably not a major factor contributing to disease progression.

### *p53*

Recent studies have detected mutations in the p53 gene in some soft tissue sarcomas,[31,32] and fibromatosis.[33] King reported the development of chondrosarcoma in patients with Li-Fraumeni syndrome implicating p53 mutation in the malignant transformation of cartilage tissue.[34] Toguchida *et al.* examined a group of sarcoma patients and found a 33% incidence of p53 mutation.[32] Wadayama *et al.* identified 25% of sarcomas having a p53 mutation, with five of 20 chondrosarcomas having mutations.[34] This finding was associated predominantly with a dedifferentiated phenotype in each case.[35] Dobashi *et al.* examined p53 expression in 16 chondrosarcoma specimens and found only two patients with over-expression, however, both of these were high-grade (III) lesions.[36] Yang *et al.* found p53 over-expression to be of no prognostic significance in a series of 54 malignant fibrous histiocytomas.[37] Nawa *et al.* reported a correlation of p53 staining with patient survival; however, in multivariate analysis this did not emerge as an independent predictor.[38] Lastly, Simms *et al.* reported strong staining in the non-cartilaginous component of dedifferentiated chondrosarcoma patients in all of the eight patients examined and only light staining in the cartilaginous component of the lesion in two patients.[39]

The experience of these investigators is consistent with the data presented in the current study which shows that four of six dedifferentiated chondrosarcomas stained positively for p53. As mentioned, this subset of patients faired poorly from a clinical standpoint. So while it seems that in these patients p53 mutations may be associated with the dedifferentiated phenotype, they are not a useful prognostic indicator of chondrosarcoma recurrence in general. It also suggests that the dedifferentiated component of chondrosarcoma should be considered a separate disease process.

### *rb-1*

The retinoblastoma gene (RB) codes for a nuclear phosphoprotein involved in cell cycle control.[40] The RB gene is mutated or absent in a variety of malignancies.[15,24,38,41–47] Monoclonal antibodies have been developed for identification of the Rb protein in paraffin-embedded tissues. Studies correlating Rb protein status and clinical outcome have demonstrated that the absence of the Rb protein can be associated with a poor prognosis[35,48] as well as resistance to some chemotherapy agents.[49] In the patients examined in the current study, 27 of 39 patients stained positively for Rb protein. There was no statistical correlation between Rb-1 staining and event-free survival.

*MIB-1*

The cell proliferation index has been determined in the past with thymidine labeling,[50] 5-bromodeoxyuridine labeling,[51] flow cytometry[52–54] and by quantitation of nucleolar organizer regions.[55] Assessment of the growth fraction may also be performed on frozen sections by Ki-67 immunostaining which reacts with a proliferation associated antigen expressed in all cells not in $G_0$ or portions of the $G_1$ phase of the cell cycle.[56,57] More recently, several antibodies have been described that demonstrate proliferating cells in formalin-fixed tissue including Ki-67, PCNA and MIB-1.[58–60] MIB-1 staining has been demonstrated to have prognostic influence in patients with breast carcinoma,[61,62] ovarian cancer,[63] but a recent study examining benign and malignant fibrohistiocytic lesions demonstrated only a limited role for MIB-1 in distinguishing between these lesions.[64] In contrast, Yang *et al.* did show that the proliferation index as determined by Ki-67 immunostaining had prognostic value in fibrous lesions.[65] Our data supported the usefulness of the proliferation index (MIB-1) as a significant prognostic indicator for chondrosarcoma: a higher proliferation index was associated with a lower event-free survival ($p > 0.01$).

DNA analysis by flow cytometry has been explored in the past as a means of predicting biologic behavior of chondrosarcoma.[66] Alho *et al.* reported a statistically significant association between malignant behavior of the tumor and aneuploid content.[67] A later report by the same senior investigator extended this analysis over a larger patient population with similar results.[67] In analysis by flow cytometry, it can be difficult to differentiate DNA ploidy abnormalities from changes in DNA content associated with the cell cycle. More recently, an independent investigator has reported that the S-phase fraction rather than aneuploidy correlates most closely with biologic behavior.[69] The number of tumors examined was small (four benign and four malignant cartilaginous tumors), but the data suggest that the proportion of cells that have entered the cell cycle was prognostically important. An alternative means of determining the fraction of cells entering the cell cycle is to examine the expression of the Ki-67 epitope. Nawa *et al.* have recently reported a correlation of MIB-1 staining with histologic grade and event-free survival.[38] Nawa *et al.* found a mean proliferation index of 3.2% for grade I lesions, 14.7% for grade II lesions and 16.4% for grade III lesions. This is consistent with the distribution of proliferation indices that was observed in the current study and indicates that the proliferative index serves as a prognostic indicator for chondrosarcoma.

It is interesting that the cell proliferation rate appears to be an important prognostic factor in patients with chondrosarcoma yet two of the proteins identified in cell cycle control do not appear to be involved. This leads one to speculate that there are additional mechanisms regulating the proliferative rate in cartilaginous tissues.

### Concluding remarks

The data presented in this report indicate that the proliferation rate of chondrosarcoma cells serves as a prognostic indicator for disease recurrence and clinical outcome. Tumors with a high rate of cell proliferation have a worse prognosis and patients afflicted with these tumors demonstrate a shorter event-free survival. MIB-1 may serve as a useful clinical adjunct to histopathologic grading and flow cytometry in the staging and treatment of patients with chondrosarcoma. Prognosis was not correlated to the level of regulatory protein products (oncogenes, Rb-1, *src*, p53) in our experience. Perhaps quantitation of other regulatory oncogenes of cell proliferation as well as markers of cell–cell cohesion or invasiveness might yield results more predictive of tumor behavior and correlate with the proliferative index. A high proliferation index at the time of biopsy may lead clinicians to consider existing adjuvant therapies as these tumors may be more responsive because of their increased rate of DNA synthesis. Alternatively, future adjuvant therapies such as immunotherapy or biologic modulation may be appropriate for these patients who are at high risk of recurrent disease.

### References

1 Mirra JM. *Bone tumors*, Philadelphia: Lea & Febiger, 1989.

2 Eriksson AI, Schiller A, Mankin HJ. The management of chondrosarcoma of bone. *Clin Orthop Related Res* 1980; 153:44–66.

3 Barnes R, Catto M. Chondrosarcoma of bone, *J Bone Joint Surg* 1966; 48B:729–33.

4 Henderson ED, Dahlin DC. Chondrosarcoma of bone: a study of 228 cases. *J Bone Joint Surg* 1963; 45A:1450–65.

5 Evans HL, Ayala AG, Romsdahl MM. Prognostic factors in chondrosarcoma of bone cancer. *Cancer* 1977; 40:818–31.

6 Wang JW, Ger LP, Shih CH, *et al.* Chondrosarcoma of bone: a statistical analysis of prognostic factors. *J Formosan Med Assoc* 1991; 90:998–1003.

7 Dahlin DC. Chondrosarcoma. A surgical and pathologic problem. *J Bone Joint Surg* 1956; 38A:1025–38.

8 Marcove RC, Huvos AG. Cartilaginous tumors of the ribs. *Cancer* 1971; 27:794.

9 Yang P, Hirose T, Hasegawa T, *et al.* Prognostic implications of the p53 protein and Ki-67 antigen immunohistochemistry in malignant fibrous histiocytoma. *Cancer* 1995; 76:618–25.

10 Kastan MB, Onyekwere O, Sidransky D, *et al.* Participation of p53 protein in the cellular response to DNA damage. *Cancer Res* 1991; 51:6304–11.

11 Lane DP. p-53 guardian of the genome. *Nature* 1992; 358:15–16.

12 Finlay CA, Hinds PW, Tan TH, *et al.* Activating mutations for transformation by p-53 produce a gene product that forms an hsc 700–p53 complex with an altered half-life. *Mol Cell Biol* 1988; 8:531–9.

13 Hesketh R. *The oncogene handbook*. London: Academic Press, 1994; 448–81.
14 Kerns BJ, Jordan PA, Faerman LL, *et al.* Determination of proliferation index with MIB-1 in advanced ovarian cancer using quantitative image analysis. *Am J Clin Pathol* 1994; 101:192–7.
15 Taylor SJ, Shalloway D. The cell cycle and c-*src*. *Curr Opin Genet Dev* 1993; 3:26–34.
16 Ewen ME, Xing Y, Lawrence JB, *et al.* Molecular cloning, chromosomal mapping and expression of the cDNA for p107, a retinoblastoma gene product-related protein. *Cell* 1991; 66:1155–64.
17 Layfield LJ, Prognostic indicators for neuroblastoma: stage, grade, DNA ploidy, MIB-1 proliferation index, p53, HER-2/neu and EGFr—a survival study. *J Surg Onc* 1995; 59:21–7.
18 Kerns BJ, Jordan PA, Moore MB, *et al.* p53 overexpression in formalin-fixed paraffin embedded tissue detected by immunohistochemistry. *J Histochem Cytochem* 1992; 40:1047–52.
19 Kerns BJ, Jordan PA, Huper G, *et al.* Assessment of c-erbB-2 amplification by immunohistochemistry in paraffin embedded breast cancer. *Mod Pathol* 1993; 6:673–8.
20 Key G, Becker MHG, Duchrow M, *et al.* New Ki-67 equivalent murine monoclonal antibodies (MIBl-3) prepared against recombinant parts of the Ki-67 antigen. *Anal Cell Pathology* 1992; 4:181–4.
21 Banks L, Matlashewski G, Crawford L. Isolation of human-p53-specific monoclonal antibodies and their use in the studies of human p53 expression. *Eur J Biochem* 1986; 159:529–34.
22 Ali AA, Marcus JN, Harvey JP, *et al.* Rb1 protein in normal and malignant human colorectal tissue and colon cancer cell lines. *FASEB J* 1993; 7:931–7.
23 Layfield LJ, Kerns BJM, Conlon DJ, *et al.* Determination of proliferation index by MIB-1 in early stage breast carcinoma using quantitative image analysis. *Breast J* 1995; 1:362–71.
24 Kaplan EL, Meier P. Non parametric estimation from incomplete observations. *J Am Stat Assoc* 1958; 53:467.
25 Gehan E. A generalized Wilcoxon test for comparing arbitrarily singly-censored samples. *Biometrika* 1965; 52:203.
26 Tanaka A, Gibbs CP, Arthan RB, *et al.* DNA sequence encodes the aminoterminal region of the human C-src protein: implications of sequence divergence among src-type kinase oncogenes. *Mol Cell Biol* 1987, 7:1978–83.
27 Soriano P, Montgomery C, Geske R, *et al.* Targeted disruption of the src protooncogene LEADS to osteoporosis in mice. *Cell* 1991; 64:693–702.
28 Hesketh R. *The oncogene handbook*. London: Academic Press 1994; 462–3.
29 Alema S, Tato F, Boettiger D. Myc and *Src* oncogenes have complimentary effects on cell proliferation and expression of specific extracellular matrix components in definitive chondroblasts. *Mol Cell Biol* 1985; 5:538–44.
30 Zhu J, Pan HO, Suzuki F, *et al.* Proto-oncogene expression in human chondrosarcoma cell line: HCS-2/8. *Jpn J Cancer Res* 1994; 85:364–71.
31 Andreasson A, Oyjord T, Hovig E. p53 abnormalities in different subtypes of human sarcomas. *Cancer Res* 1993; 53:468–71.
32 Toguchida J, Yamaguchi T, Richie B. Mutation spectrum of the p53 gene in bone and soft tissue sarcomas. *Cancer Res* 1992; 52:6194–9.
33 Oshiro Y, Fukuda T, Tsuneyoshi M. Fibrosarcoma versus fibromatosis and cellular nodular fascitis: a comparative study of their proliferative activity using proliferating cell nuclear antigen. DNA flow cytometry, and p53. *Am J Surg Path* 1994; 18:712–19.
34 King P, Craft AW, Malcolm AJ. p53 expression in three separate tumors from a patient with Li-Fraumeni's syndrome. *J Clin Pathol* 1993; 46:676–7.
35 Wadayama B, Toguchida J, Yamaguchi T, *et al.* p53 expression and its relationship to DNA alterations in bone and soft tissue sarcomas. *Br J Cancer* 1993; 68:1134–9.
36 Dobashi Y, Sugimura H, Sato A, *et al.* Possible association of p53 overexpression and mutation with high-grade chondrosarcoma. *Diagn Mol Pathol* 1993; 2:257–63.
37 Yang P, Hirose T, Hasegawa T, *et al.* Prognostic implications of the p53 protein and Ki-67 antigen immunohistochemistry in malignant fibrous histiocytoma. *Cancer* 1995; 76:618–25.
38 Nawa G, Ueda T, Mori S, *et al.* Prognostic significance of Ki67 (MIB1) proliferation index and p53 over-expression in chondrosarcomas. *Int J Cancer* l996; 69:86–91.
39 Simms WW, Ordonez NG, Jonston D, *et al.* p53 expression in dedifferentiated chondrosarcoma. *Cancer* 1995; 76:223–7.
40 Geradts J, Hu SX, Lincoln CE, *et al.* Aberrant Rb gene expression in routinely processed, archival tumor tissues determined by three different Rb antibodies. *Int J Cancer* 1994; 58:161–7.
41 Brickell PM. The 60c-*src* family of protein-tyrosine kinases: structure, regulation, and function. *Crit Rev Oncog* 1992; 3:401–46.
42 Shimizu E, Coxon A, Oherson GA, *et al.* RB protein status and clinical correlation from 171 cell lines representing lung cancer, extrapulmonary small cell carcinoma, and mesothelioma. *Oncogene* 1994; 9:2441–8.
43 Wadayama B, Toguchida J, Shimizu T, *et al.* Mutation spectrum of the retinoblastoma gene in osteosarcomas. *Cancer Res* 1994; 54:3042–8.
44 Kim MS, Li SL, Bertolami CN, *et al.* State of p53, Rb and DCC tumor suppressor genes in human oral cancer cell lines. *Anticancer Res* 1993; 13:1405–13.
45 Reissman PT, Koga H, Takahashi R, *et al.* Inactivation of the retinoblastoma susceptibility gene in non-small cell lung cancer. The Lung Cancer Study Group. *Oncogene* 1993; 8:1913–19.
46 Trudel M, Mulligan L, Cavenee W, *et al.* Retinoblastoma and p53 gene product expression in breast carcinoma: immunohistochemical analysis and clinicopathologic correlation. *Hum Pathol* 1992; 23:1388–94.
47 Spandidos DA, Karaissofidi H, Malliri A, *et al.* Expression of MS, Rbl and p53 proteins in human breast cancer. *Anticancer Res* 1992; 12:81–9.
48 Resiman PT, Koga, H, Takahashi R, *et al.* Inactivation of the retinoblastoma susceptibility gene in non-small cell lung cancer. The Lung Cancer Group. *Oncogene* 1993; 8:1913–19.
49 Shimizu E, Coxon A, Oherson GA, *et al.* Rb protein status and clinical correlation from 171 cell lines representing lung cancer, extrapulmonary small cell carcinoma, and mesothelioma. *Oncogene* 1994; 9:2441–8.
50 Karmel OW, Franklin WA, Ringus JC, *et al.* Thymidine labeling index and Ki-67 growth fractions in lesions in the breast. *Am J Pathol* 1989; 34:107–13.
51 Saski K, Matsumura T, Tsuji T, *et al.* Relationship between labeling indices of Ki-67 and BrdUrd in human malignant tumors. *Cancer* 1988; 62:989–93.
52 Kennedy JC, El-Badawy N, DeRose PB, *et al.* Comparison of cell proliferation in breast carcinoma using image analysis (Ki-67) and flow cytometric systems. *Anal Quant Cytol Histol* 1992; 14:304–11.

53 Isoloa JJ, Helin JH, Helle MJ, *et al*. Evaluation of cell proliferation in breast carcinoma. Comparison of Ki-67 immunohistochemical study. DNA flow cytometric analysis, and mitotic count. *Cancer* 1990; 65:1180–4.
54 Dawson AE, Norton JA, Weinberg DS. Comparative assessment of proliferation and DNA content in breast carcinoma by image analysis and flow cytometry. *Am J Pathol* 1990; 136:1115–24.
55 Ohno T, Tanaka T, Takeuchi S, *et al*. Nucleolar organizer regions in bone tumors. *Clin Orthol Related Res* 1991; 272:287–91.
56 Gerdes J, Schwab U, Lemke H, *et al*. Production of a mouse monoclonal antibody reactive with a human nuclear antigen associated with cell proliferation. *Int J Cancer* 1983; 31:13–20.
57 Gerdes J, Lemke H, Baisch H, *et al*. Cell cycle analysis of a cell proliferation associated human nuclear antigen defined by the monoclonal antibody Ki-67. *J Immunol* 1984; 133:1710–15.
58 Cattoretti G, Becker MH, Key G, *et al*. Monoclonal antibodies against recombinant parts of the Ki-67 antigen (MIB 1 and MIB 3) detect proliferating cells in microwave-processed formalin-fixed paraffin sections. *J Pathol* 1992; 168:357–63.
59 McCormick D, Chong H, Hobbs C, *et al*. Detection of the Ki-67 antigen in fixed and wax-embedded sections with the monoclonal antibody MIB1. *Histopathology* 1993; 22:355–60.
60 McCormick D, Yu C, Hobbs C. The relevance of antibody concentration to the immunohistological quantification of cell proliferation-associated antigens. *Histopathology* 1993; 22:543–7.
61 Pinder SE, Wencyk P, Sibbering DM, *et al*. Assessment of the new proliferation marker MIB-1 in breast carcinoma using image analysis: associations with other prognostic factors and survival. *Br J Cancer* 1995; 71:146–9.
62 Pich A, Margaria E, Chiusa L. Proliferative activity is a significant prognostic factor in male breast carcinoma. *Am J Path* 1994; 145:481–9.
63 Kerns BJ, Jordan PA, Faerman LL, *et al*. Determination of proliferation index with MIB-1 in advanced ovarian cancer using quantitative image analysis. *Am J Clin Path* 1994; 101:192–7.
64 Oshiro Y, Fukuda T, Tsuneyoshi M. Atypical fibroxanthoma versus benign and malignant fibrous histiocytoma. A comparative study of their proliferative activity using MIB-1, DNA flow cytometry, and p53 immunostaining. *Cancer* 1995; 75:1128–34.
65 Yang P, Hirose T, Hasegawa T, *et al*. Prognostic implications of the p53 protein and Ki-67 antigen immunohistochemistry in malignant fibrous histiocytoma. *Cancer* 1995; 76:618–25.
66 Alho A, Connor JF, Mankin HJ, *et al*. Assessment of cartilage tumors using flow cytometry, *J Bone Joint Surg* 1983; 65A:779–85.
67 Alho A, Connor JF, Mankin HJ, *et al*. Assessment of malignancy of cartilaginous tumors using flow cytometry. *J Bone Joint Surg* 1983; 65A:779–85.
68 Kolettis GJ, Li XQ, Gebhendt MC, *et al*. Flow cytometry as a predictor of biological behavior for chondrosarcomas. *Orthop Trans* 1994; 18:589–90.
69 Alho A, Skjeldal S, Melvik JE, *et al*. The clinical importance on DNA synthesis and aneuploidy in bone and soft tissue tumors. *Anticancer Res* 1993; 13:2383–8.
